# Vimentin Tail Segments Are Differentially Exposed at Distinct Cellular Locations and in Response to Stress

**DOI:** 10.3389/fcell.2022.908263

**Published:** 2022-06-08

**Authors:** Irene Lois-Bermejo, Patricia González-Jiménez, Sofia Duarte, María A. Pajares, Dolores Pérez-Sala

**Affiliations:** Department of Structural and Chemical Biology, Centro de Investigaciones Biológicas Margarita Salas, CSIC, Madrid, Spain

**Keywords:** lipoxidation, posttranslational modifications, vimentin tail, intermediate filament, electrophilic stress, vimentin tail phosphorylation, vimentin reorganization

## Abstract

The intermediate filament protein vimentin plays a key role in cell signaling and stress sensing, as well as an integrator of cytoskeletal dynamics. The vimentin monomer consists of a central rod-like domain and intrinsically disordered head and tail domains. Although the organization of vimentin oligomers in filaments is beginning to be understood, the precise disposition of the tail region remains to be elucidated. Here we observed that electrophilic stress-induced condensation shielded vimentin from recognition by antibodies against specific segments of the tail domain. A detailed characterization revealed that vimentin tail segments are differentially exposed at distinct subcellular locations, both in basal and stress conditions. The 411–423 segment appeared accessible in all cell areas, correlating with vimentin abundance. In contrast, the 419–438 segment was more scantily recognized in perinuclear vimentin and lipoxidative stress-induced bundles, and better detected in peripheral filaments, where it appeared to protrude further from the filament core. These differences persisted in mitotic cells. Interestingly, both tail segments showed closer accessibility in calyculin A-treated cells and phosphomimetic mutants of the C-terminal region. Our results lead us to hypothesize the presence of at least two distinct arrangements of vimentin tail in cells: an “extended” conformation (accessible 419–438 segment), preferentially detected in peripheral areas with looser filaments, and a “packed” conformation (shielded 419–438 segment), preferentially detected at the cell center in robust filaments and lipoxidative stress-induced bundles. These different arrangements could be putatively interconverted by posttranslational modifications, contributing to the versatility of vimentin functions and/or interactions.

## Introduction

Vimentin is a type III intermediate filament protein that typically forms an extended network in the cytoplasm of mesenchymal cells, maintaining a close interplay with actin and tubulin structures. Vimentin is involved in numerous functions in physiology, and in the response to stress. Thus, vimentin plays a key role in the positioning and homeostasis of cellular organelles, cell mechanical properties, cell migration and division (Ivaska et al., 2007; Minin and Moldaver, 2008; Hol and Capetanaki, 2017). Pathophysiological situations associated with vimentin overexpression or dysfunction include cancer, fibrosis, and inflammation (Satelli and Li, 2011; Li et al., 2021; dos Santos et al., 2015). Interestingly, vimentin can also be encountered in the extracellular milieu where it has been involved in cell migration, intercellular communication and pathogen infection (reviewed in Ramos et al., 2020).

Vimentin filaments are extraordinarily versatile and complex structures, which adapt to various cellular situations and stress through a finely tuned regulation (Snider and Omary, 2014; Viedma-Poyatos et al., 2020). Knowledge from *in vitro* experiments has led to propose an assembly sequence consisting in the parallel association of two vimentin monomers into dimers, which then would join in a staggered antiparallel manner to give rise to a tetrameric structure. Several of these tetramers, typically eight in most models, associate laterally to form a unit length filament or “ULF,” and end to end connection of many ULF conforms the filaments (Herrmann and Aebi, 2016; Eldirany et al., 2021). As described, this process could result in homogeneous, rope-like structures. However, in cells, filaments coexist with robust bundles, unraveled structures, and oligomeric non-filamentous vimentin, all of which can swiftly arise in response to various stimuli. Moreover, even single filaments are highly polymorphic and several assembly modes, involving different number of tetramers and interactions, have been identified through high resolution cryo-electron microscopy studies of straight sections of cellular filaments (Eibauer et al., 2021).

This versatility is supported by the high diversity of vimentin proteoforms. Nearly 150 modifications of human vimentin appear listed in PhosphoSite v6.6.0.4 (www.phosphosite.org), which could occur in different combinations, giving rise to an extraordinary variety of species differing in their assembly properties. Moreover, vimentin is the target for numerous non-enzymatic modifications by structurally diverse reactive species, which increase its polymorphism (Viedma-Poyatos et al., 2020; Griesser et al., 2021). The structure of the vimentin monomer comprises a central rod domain, which is mainly α-helical and integrated by several coils, and N-terminal (head) and C-terminal (tail) domains of intrinsically disordered structure. The flexible head domain has been reported to fold back onto the rod establishing interactions with coil 1A (Aziz et al., 2009; Aziz et al., 2010). In addition, interactions of the head and coil 1A have been reported to connect adjacent “protofibrils” (octameric strands) within assembled filaments (Eibauer et al., 2021). Moreover, the head domain is a key regulator of filament disassembly since it harbors at least eleven phosphorylation sites that are key for vimentin reorganization in mitosis (Matsuyama et al., 2013).

On the other hand, the structure and functions of the intrinsically disordered tail domain, comprising amino acids 412–466, are less understood. Vimentin tails are thought to protrude out of the rod forming a brush-like structure around the filament core (Kornreich et al., 2015). This seems to be in agreement with immunofluorescence analyses carried out by labeling single filaments with antibodies against the rod and the tail domain, which revealed a “cloud” of C-terminal segments around the compact rod domains (Herrmann and Aebi, 2016). In turn, the disposition of the tails in the brush structure could adopt different conformations due to the interaction between two conserved sequences, i.e. the β-turn formed by residues TRDG and the so-called “epsilon site” on the distal part of the rod comprising residues 364–416 (Kouklis et al., 1991; Hess et al., 2013). These complementary sites appear to associate during filament assembly forming a loop that would prevent filament aggregation and regulate filament thickness (Kouklis et al., 1991). Consistent with this, tailless vimentin can form nearly normal filaments *in vitro*, although wider than wild type filaments and with a higher number of tetramers per cross-section (Eckelt et al., 1992; Brennich et al., 2019). Importantly, the tail domain plays a critical role in vimentin distribution in cells; it acts as a cytoplasmic localization signal and is critical for vimentin intertwining with and modulation of the actin cortex in mitosis (Duarte et al., 2019). Moreover, tailless vimentin forms thick bundles that remain in the proximity of the nucleus causing mitotic alterations (Duarte et al., 2019), and, if present in excess over the full-length form, deeply disrupts the filament network provoking a perinuclear collapse.

In cells, diverse stimuli and types of stress induce fast vimentin remodeling. Vimentin phosphorylation can induce disassembly, for instance, during mitosis (Yamaguchi et al., 2005; Sihag et al., 2007), whereas modifications of its single cysteine residue (C328) in response to oxidative or lipoxidative challenges induce disruption of the network (Pérez-Sala et al., 2015; Mónico et al., 2019). Nevertheless, how these changes affect the tail domain, and *vice versa*, is not completely understood. In contrast to the head domain, fewer studies are available on the consequences of tail domain phosphorylation. Importantly, the tail domain contains several protease cleavage sites, including some for viral proteases (Singh and Arlinghaus, 1989; Shoeman et al., 1990) which cause vimentin perinuclear collapse (Duarte et al., 2019), an effect that has been hypothesized to contribute to the nuclear translocation and import of the viral genome (Thomas et al., 1996; Zhang et al., 2020).

We have previously shown that electrophilic stress induces drastic vimentin remodeling with condensation of filaments in thick juxtanuclear bundles (Pérez-Sala et al., 2015; Mónico et al., 2019). In this work, we attempted to monitor vimentin reorganization by immunofluorescence with antibodies against the tail domain. Given the complexity of the results obtained, additional antibodies with epitopes mapping to consecutive overlapping segments of the vimentin tail were employed to characterize this effect. We have observed that the accessibility of the various tail epitopes varies with the spatial distribution of vimentin in cells under control conditions and with its reorganization in response to stress. Our results are consistent with a dynamic role of the vimentin tail, which could hypothetically influence vimentin assembly and/or interactions in a spatio-temporal manner.

## Materials and Methods

### Reagents

Anti-vimentin antibodies used included mouse monoclonal V9 (Santa Cruz Biotechnology, sc-6260), mouse monoclonal E5 (Santa Cruz Biotechnology, sc-373717), rabbit polyclonal “C-term” (RayBiotech, 102-15110), goat polyclonal “C-end” (Everest Biotech, United Kingdom, EB11207) and “N-term” (Cell Signaling Technology, D21H3), as well as the V9 and E5 conjugates with Alexa Fluor 488 or Alexa Fluor 546, and Alexa Fluor 647 conjugated D21H3, as specified where it corresponds. Anti-actin antibody was from Sigma (A2066). Several secondary antibodies were used: anti-mouse, anti-rabbit and anti-goat immunoglobulins, conjugated with Alexa Fluor 488, 546 or 647, all obtained from Molecular Probes. HRP-conjugated anti-mouse, anti-rabbit and anti-goat antibodies were from Dako. 4,6-diamidino-2-phenylindole (DAPI), Phalloidin-TRITC and acrolein were from Sigma. 4-hydroxynonenal (HNE) was from Cayman Chemical. Calyculin A was from Santa Cruz Biotechnology.

### Cell Culture and Treatments

Human adrenal carcinoma cells SW13/cl.2 were a generous gift from Dr. A. Sarriá (University of Zaragoza, Spain). The human primary fibroblasts AG10803 used in this study were from the National Institute of General Medical Sciences (NIGMS) Human Genetic Cell Repository at the Coriell Institute for Medical Research (Candem, NJ, United States). Vero cells, fibroblast-like green monkey kidney cells, were obtained from the collection of Centro de Investigaciones Biológicas Margarita Salas (CIB-CSIC, Madrid). HeLa human cervical carcinoma cells were from the American Type Cell Culture Collection (ATCC). All the cell lines described were cultured in DMEM, supplemented with 10% (v/v) Fetal Bovine Serum (FBS, Biowest), penicillin (100 U/ml) and streptomycin (100 μg/ml) (Invitrogen), except for AG10803 fibroblasts, which were cultured in 20% (v/v) FBS. Cells were kept at 37°C in a humid atmosphere enriched with 5% CO_2_. For electrophilic stress studies, cells were treated with vehicle (DMSO) or electrophilic lipids, HNE (10 μM) for 4 h or acrolein (10 μM) for 2 h in serum-free medium. Treatment with calyculin A (20 nM) was performed for 20 min in serum-free medium.

### Plasmids and Transfections

Plasmids used in this study include pIRES-DsRed-Express2-vimentin (RFP//vimentin), for expression of untagged vimentin wt, and pEGFP-C1-vimentin wt, described previously (Pérez-Sala et al., 2015), and pmCherry-C1-vimentin wt (Genecopoeia). For cotransfection of plasmids expressing tagged and untagged vimentin constructs a 2:8 DNA ratio was used. Truncated forms of vimentin were expressed from plasmids RFP//vim(1–411), RFP//vim(1–423), RFP//vim(1–448) and RFP//vim(1–459), obtained in our laboratory by introduction of premature stop codons in the original RFP//vimentin plasmid, as previously described (Duarte et al., 2019). Vimentin phosphomimetic constructs, where various serine residues were substituted by aspartic acid (S325D, S412D, S419D), were obtained by mutagenesis of the corresponding RFP//vimentin S➤A mutant plasmids, using the Nzytech mutagenesis kit and the primers specified in Table 1. Numbering of vimentin residues in this study considers the initial methionine as position 1. Exogenous expression of vimentin was induced by transfection of the corresponding plasmids into cells in culture. SW12/cl.2 cells at 70% confluency were transfected with 1 µg of DNA, using Lipofectamine 2000 (Invitrogen), following manufacturer’s indications. Cells were fixed for further processing 48 h after transfection.

**TABLE 1:** Primers used in this study.

Mutant	Primers	
	Forward	Reverse
S325D	GGA​GAC​AGG​TGC​AGG​ACC​TCA​CCT​GTG​AAG​TGG	CCA​CTT​CAC​AGG​TGA​GGT​CCT​GCA​CCT​GTC​TCC
S412D	GCG​AGG​AGA​GCA​GGA​TTG​ATC​TGC​CTC​TTC​C	GGA​AGA​GGC​AGA​TCA​ATC​CTG​CTC​TCC​TCG​C
S459D	GGT​TAT​CAA​CGA​AAC​TGA​TCA​GCA​TCA​CGA​TGA​CC	GGT​CAT​CGT​GAT​GCT​GAT​CAG​TTT​CGT​TGA​TAA​CC

### Cell Lysis and Western Blot

Cells were lysed in 50 mM Tris-HCl pH 7.5, 0.1 mM EDTA, 0.1 mM EGTA, 0.1 mM β-mercaptoethanol, 0.5% (w/v) SDS, 50 mM sodium fluoride, 0.1 mM sodium orthovanadate and protease inhibitors: 2 μg/ml leupeptin, 2 μg/ml aprotinin, 2 μg/ml trypsin inhibitor and 1.3 mM Pefablock. Moreover, complete lysis was ensured by passing the cells through a narrow needle (26^1/2^G). The cell lysate was subsequently centrifuged (10,000 g, 4°C, 5 min) and the protein concentration of the supernatant was measured with the bicinchoninic acid (BCA) protein quantification kit (ThermoFischer Scientific). Samples (30 μg of total protein) were denatured in Laemmli buffer (80 mM Tris-HCl pH 6.8, 2% (w/v) SDS, 10% (v/v) glycerol, 5% (v/v) β-mercaptoethanol, 0.15% (w/v) bromophenol blue), during 5 min at 95°C, and loaded onto 12.5% (w/v) SDS-polyacrylamide gels. Proteins were transferred to Immobilon-P membranes (Millipore) using a Transblot semidry transfer unit (Bio-Rad), following a Tris-glycine methanol three-buffer system protocol, as indicated by the manufacturer. Membranes were blocked in 2% (w/v) low-fat powdered milk in 20 mM Tris-HCl pH 7.5, 500 mM NaCl, 0.05% (v/v) Tween 20 (T-TBS), incubated with primary antibodies for 2 h (V9, E5, N-term, anti-actin) or overnight (C-term, C-end), and for 1 h with secondary HRP-conjugated antibodies after washing. Antibodies were diluted in 1% (w/v) bovine serum albumin (BSA) in T-TBS. Signal on membranes was developed with ECL reagents (GE Healthcare) and captured on photographic films (Agfa).

### Immunofluorescence

Cells were fixed in 4% (w/v) paraformaldehyde (PFA) for 25 min, and permeabilized with 0.1% (v/v) Triton X-100 in PBS for 20 min prior to being blocked in 1% (w/v) (BSA) in PBS for 1 h. Samples were incubated with either primary and secondary antibodies or with the fluorophore-conjugated primary antibodies for 1 h per antibody, previously diluted (1:200) in 1% (w/v) BSA in PBS. Nuclei were counterstained with DAPI (3 μl/ml) for 15 min, and samples were mounted in Fluorsave (Calbiochem) medium. All incubations were carried out at room temperature. For the V9 and E5 antibodies, controls of their performance in different incubation orders and immunostaining protocols were carried out to verify the results. Fixation with methanol was carried out at −20°C for 10 min and no permeabilization step was required; protocol was continued from the blocking step onwards as described above.

### Fluorescence Microscopy

Leica TCS SP5 and SP8 confocal microscopes were used to acquire images of fixed samples with a 63× objective. Single confocal sections were taken every 0.5 µm. For comparison of the signals from pairs of antibodies, images were acquired by adjusting the intensity of each channel precisely below the saturation point for each field of view, using the LUT command. Lightning deconvolution module was used together with SP8 confocal imaging, when thus stated. Superresolution microscopy by Stimulated Emission Depletion (STED) was performed in a Leica TCS SP8 microscope, with a 3X STED module. The figures show single z-planes for individual channels, their overlay or z-projections as indicated.

### Image Analysis

Images were analyzed with LAS X (Leica Microsystems) or ImageJ software. Surface plots and orthogonal projections were obtained using LAS X software. Plot profiles across individual cells were done in ImageJ for individual channels and the ratio between signals was calculated, when indicated. The recognition pattern of vimentin by the various antibodies tested was assessed through the ratio between the fluorescent intensities of the signals of selected pairs of antibodies, at an area in the vicinity of the plasma membrane. Mean values, normalized per area, allowed to ascertain which antibody signal was most prevalent at the periphery. To assess the coverage of tagged vimentin by E5 and V9 antibodies, the accessibility of E5 and V9 epitopes was determined as the colocalization (Coloc2 plugin, ImageJ) in individual cells between the signal from these antibodies and that from the GFP- or m-Cherry tagged vimentin. Colocalization is expressed as Pearson’s R coefficient and colocalization masks were obtained from the colocalization colormap plugin (ImageJ). Filament width in superresolution images was measured as the full width at half maximum (FWHM) distance in filaments with comparable intensity of V9 and E5 signals. The ratio between the width determined by E5 and V9 was calculated for normalization.

### Model Building

The hypothetical model was built based on the structure for vimentin available in AlphaFold (alphafold.ebi.ac.uk, structure ID P08670). Such structure was imitated in Inkscape software and was considered as the most compact conformation, according to the structures proposed in this study. Changes in the conformation of vimentin, as presented hereafter, were represented as opening stages from the original one. This model intends to portray the conclusions drawn from this study, but shall not be taken as an exact representation of reality.

### Statistical Analysis

All experiments were performed at least three times. Data were analyzed using GraphPad Prism software. Antibody recognition patterns were compared by the mean ratio between pairs of antibodies. Individual ratios were deemed significant by comparison to value 1, corresponding to equal intensities of antibodies, by one sample *t*-test (α = 0.05). Ratios were compared among themselves through one-way ANOVA followed by Tukey test (α = 0.05). Colocalization was defined by Pearson’s R coefficient and mean values were compared through unpaired Student’s t-test. Filament width was expressed as the ratio between V9 and E5 FWHM and mean value was compared to value 1, corresponding to equal widths (one sample *t*-test, α = 0.05).

## Results

### Electrophilic Stress-Induced Condensation of Vimentin Hampers Recognition of the Protein by an Anti-C-Terminal Domain Antibody

**FIGURE 1:**
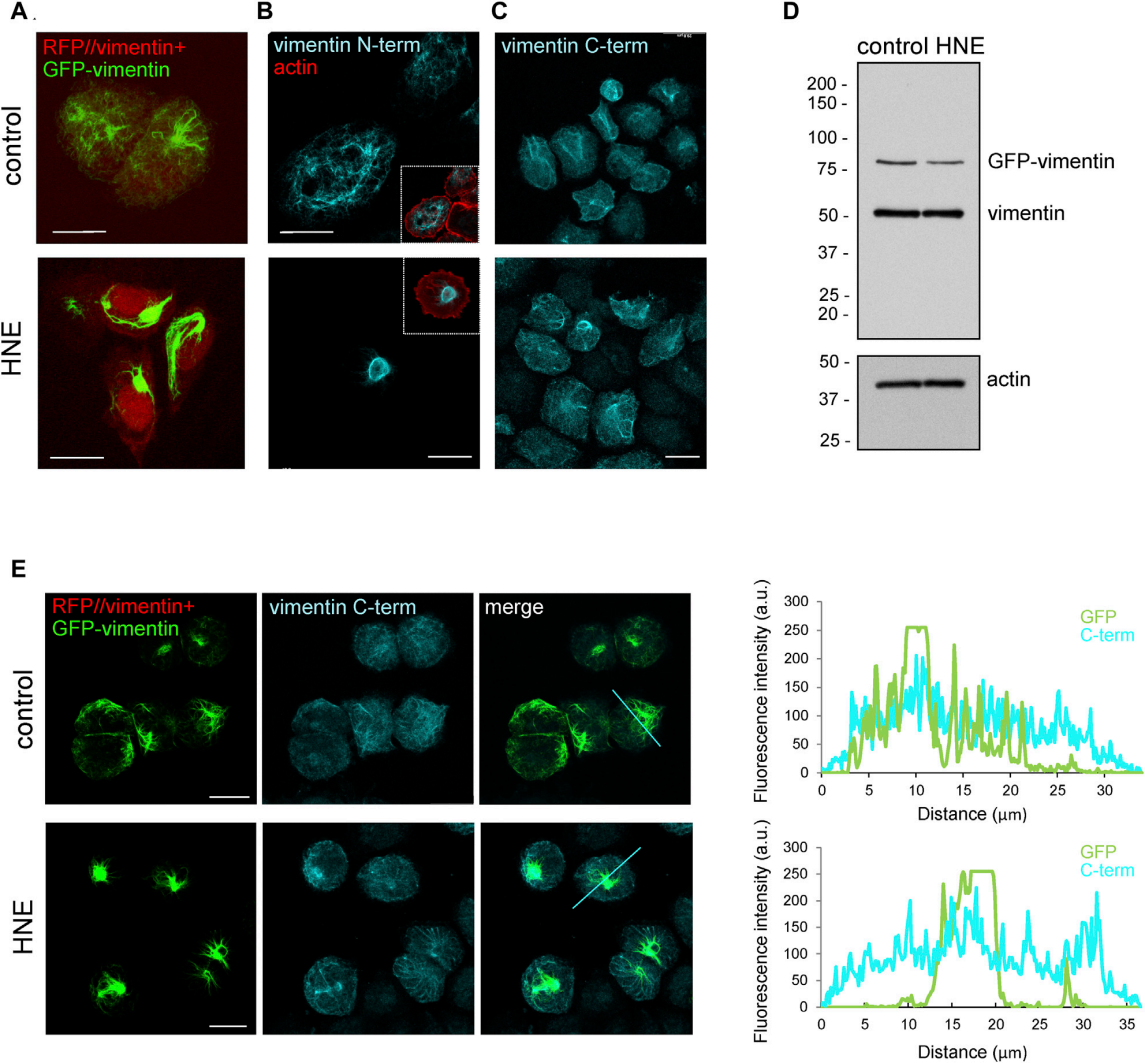
Vimentin juxtanuclear accumulations induced by treatment with HNE are poorly recognized by an anti-C-terminal antibody. SW13/cl.2 cells stably transfected with RFP//vimentin wt, coding for untagged vimentin and RFP as separate products, plus GFP-vimentin wt were treated with vehicle or 10 μM HNE for 4 h. Formation of HNE-induced vimentin accumulations was assessed by **(A)** direct fluorescence microscopy (green, GFP-vimentin; red, RFP background indicative of transfection), **(B)** immunofluorescence with an anti-N-terminal vimentin antibody, and **(C)** immunofluorescence with an anti-C-terminal vimentin antibody (C-term). In **(B)**, the cell contour is marked by staining of cortical actin with phalloidin. **(D)** Cells expressing RFP//vimentin wt plus GFP-vimentin wt were treated with vehicle or HNE and cell lysates were analyzed by western blot with V9 anti-vimentin antibody. The position of the molecular weight standards (in kDa) is shown at the left of the blots. **(E)** Cells stably transfected as in **(A)** were treated with vehicle or HNE and subjected to immunofluorescence with anti-C-terminal antibody (C-term). Vimentin distribution was monitored by confocal fluorescence microscopy. Maximal z-projections are shown. Right panels show the fluorescence intensity profiles for vimentin detected by GFP fluorescence and by immunofluorescence with the C-term antibody along the lines marked in the merged image. Scale bars, 20 μm.

The cellular vimentin network is highly responsive to electrophilic stress. We have previously reported that the reactive lipid mediator 4-hydroxynonenal (HNE) induces a marked condensation of vimentin filaments at a juxtanuclear location, forming aggresome-like structures (Pérez-Sala et al., 2015). Here, we attempted to monitor this reorganization by transfection with fluorescent fusion constructs, as well as by immunofluorescence with antibodies against different domains of the protein (Figure 1). HNE-induced vimentin accumulations were clearly detected in cells cotransfected with RFP//vimentin, for expression of the untagged protein, together with a small proportion of GFP-vimentin fusion construct to light up the vimentin network (Figure 1A), as well as by immunofluorescence with an antibody against the vimentin N-terminus (Figure 1B). Unexpectedly, aggresome-like accumulations could not be properly detected with an antibody against the vimentin segment spanning residues 430–457, located in the tail domain (C-term antibody) (Figure 1C). This lack of recognition was not due to vimentin degradation with loss of the C-terminal epitope as indicated by western blot (Figure 1D), which showed the same construct size in lysates from control and HNE-treated cells. The poor detection of HNE-induced vimentin condensations by immunofluorescence with the C-term antibody was confirmed by simultaneously monitoring the GFP-vimentin construct (Figure 1E). This revealed that the C-term antibody displayed a poor recognition, not only of vimentin accumulations in HNE-treated cells, but also of vimentin present in robust filaments or bundles in control cells, while detecting more scattered vimentin structures, as reflected in the plots of the intensity profiles (Figure 1E, right panels). Thus, these observations indicate that in certain vimentin structures the tail domain is shielded from recognition by the C-term antibody. To get further insight into this effect we next assessed the performance of additional antibodies directed against the tail domain.

### Vimentin Tail Epitopes Show Variable Accessibility Across the Cytoplasm

**FIGURE 2:**
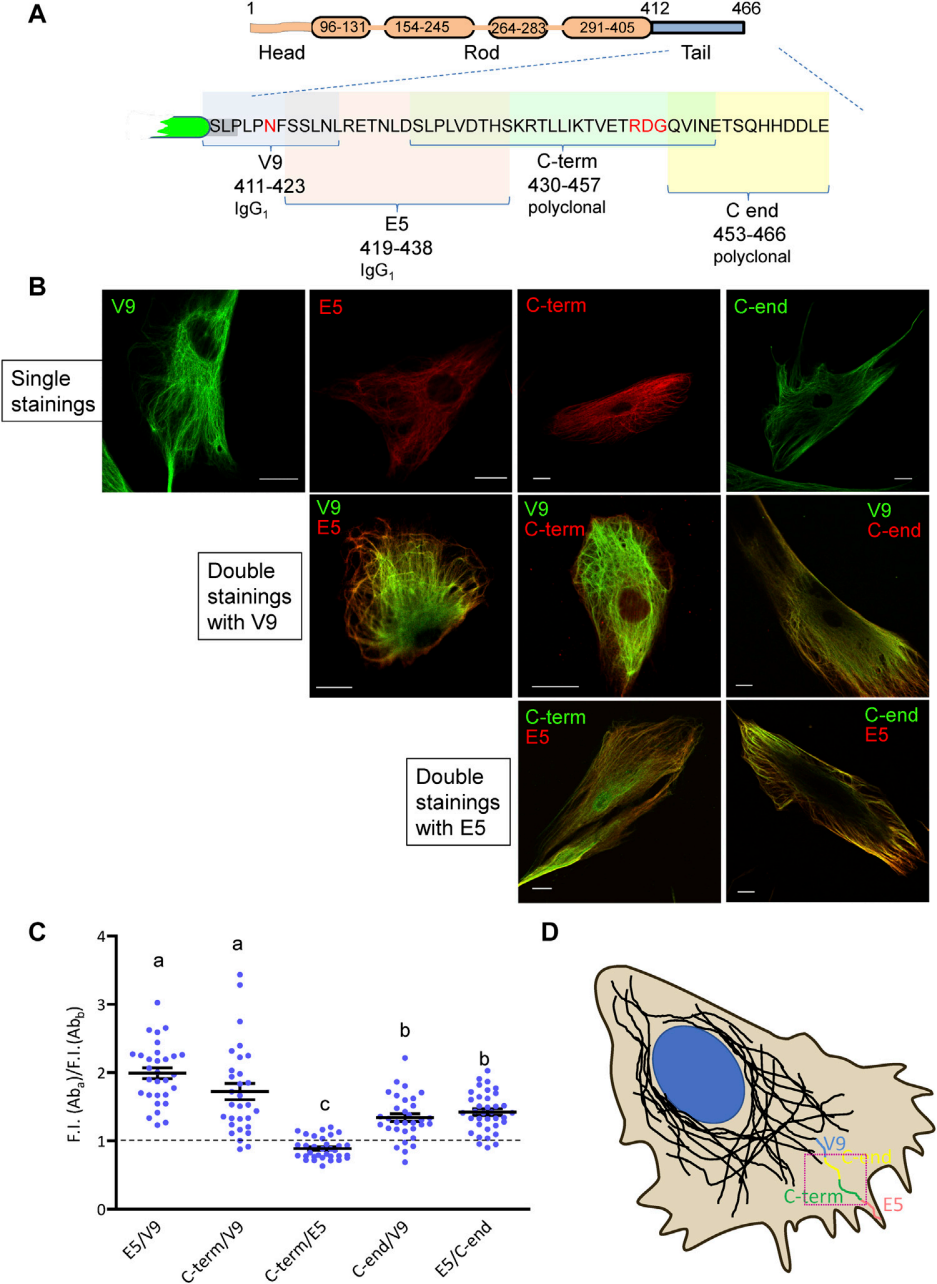
Recognition of vimentin by several antibodies with epitopes mapping at the tail domain. **(A)** Schematic representation of vimentin structure and the tail domain showing the epitopes of the antibodies used for detection. **(B)** Primary human dermal fibroblasts were subjected to immunofluorescence with the indicated antibodies, alone or in combinations, as indicated. Overlays of single z sections are shown. **(C)** Quantification of the ratio between the fluorescence intensity of pairs of antibodies at the cell periphery. For selected ROIs close to the plasma membrane, as illustrated by the red square in **(D),** fluorescence intensity was measured for both channels, and the ratio, calculated (*n* ≥ 30 for each pair). All results proved significant when means were compared to a theoretical value of 1 (corresponding to the same fluorescent intensity for both channels) through one sample *t*-test (α = 0.05). Significance among all ratios is shown in a letter-code; non-significant differences, indicated with the same letter, were only registered between pairs E5/V9—“C-term”/V9 and “C-end”/V9—E5/“C-end.” Statistical analysis was performed through 1-way ANOVA (α = 0.05), followed by Tukey test. **(D)** Schematic representation of the recognition pattern for each antibody at the periphery of cells, arranged according to their maximum reach, under non-saturation fluorescent intensity conditions. Scale bars, 20 µm.

In order to understand the variable accessibility of vimentin tail segments in wild-type cells, we analyzed its detection by antibodies recognizing consecutive segments of the tail sequence. The scheme in Figure 2A depicts a simplified view of the vimentin monomer with its head, rod and tail domains, the sequence of the latter displayed in full to show the location of the epitopes of the various antibodies used. All antibodies detected vimentin by immunofluorescence in human primary dermal fibroblasts when used individually (Figure 2B). The recognition of vimentin by different combinations of these antibodies was then studied by adjusting the signals from all antibodies to obtain maximal, though non-saturated intensities. Remarkably, we observed that the V9 antibody (epitope 411–423) yielded an intense signal throughout most of the cytoplasm and above all in the robust filaments located near the perinuclear area (Figure 2B). In contrast, both the E5 (419–438) and the C-term (430–457) antibodies appeared to preferentially recognize vimentin located at the cell periphery (Figure 2B). Interestingly, signals from E5 and C-term antibodies gave a high degree of overlap, with a slightly more frequent preferential E5 staining in peripheral filaments. Lastly, the C-end antibody yielded a signal that was proportionally higher than that of V9 at peripheral filaments but lower than that of E5. To quantify these observations, the ratios between the fluorescence intensities obtained with the different pairs of antibodies at peripheral areas of the cell (see defined ROI in Figure 2D) were calculated (Figure 2C). These results clearly showed that the C-term, E5 and C-end antibodies selectively recognized peripheral filaments with respect to V9, i.e., showed a poorer recognition of filaments at the central region of the cell. These data led us to propose a scheme according to which, the antibodies against the different segments of the tail domain would preferentially recognize vimentin at different distances from the cell center to the cell periphery in the following order: V9 (411–423), C-end (453–466), C-term (430–457) and E5 (419–438), in this cell type (Figure 2D).

**FIGURE 3:**
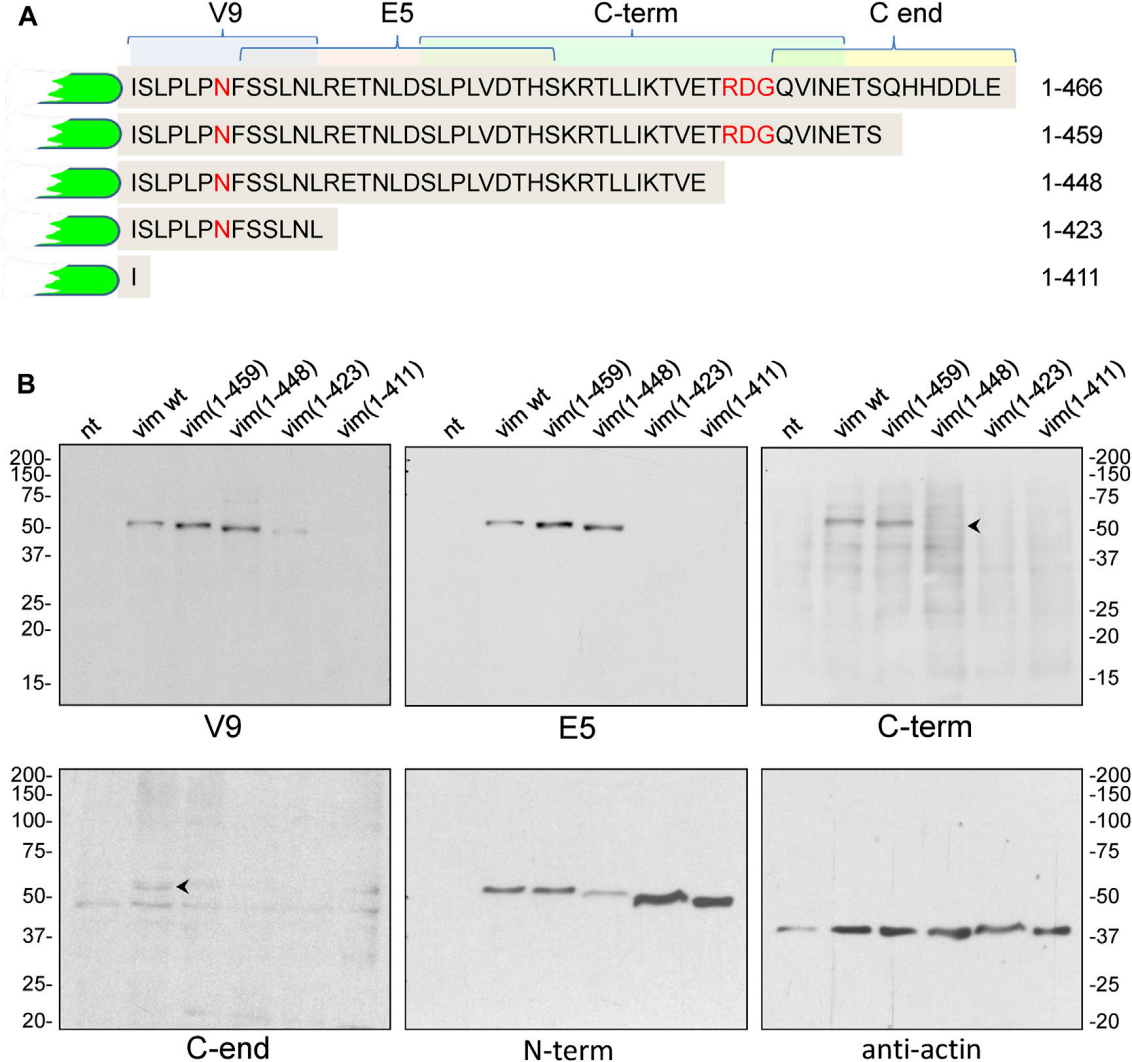
Analysis of the recognition of vimentin full length and C-terminal truncated mutants by antibodies against different epitopes of the tail domain. **(A)** Schematic of the tail sequences of the truncated vimentin mutants, indicating the epitopes recognized by the antibodies used in this study. **(B)** SW13/cl.2 cells were transfected with vimentin full length or the indicated truncated mutants. Cell lysates were analyzed by SDS-PAGE and western blot with the antibodies specified. Antibodies against vimentin N-terminus (N-term) and anti-actin were used as controls. The position of the molecular weight standards (in kDa) is shown at the left and right of the blots.

As a control for the specificity of these antibodies, we explored their ability to recognize several truncated vimentin mutants, spanning the indicated segments of the tail domain (Figure 3A), by western blot. The V9 monoclonal antibody recognized all constructs except vim(1–411), that lacks the whole tail and therefore, the V9 epitope (Figure 3B). In turn, E5 (epitope spanning the 419–438 segment), recognized all constructs except vim(1–411) and vim(1–423). The C-term antibody, raised against the 430–457 peptide, showed marginal or no recognition of the vim(1–448) construct, which indicates that the distal sequence of this peptide is necessary for antibody binding. Finally, an antibody against the last fourteen residues (C-end antibody) only recognized full-length vimentin. Nevertheless, the performance of the last two antibodies in western blot was not optimal. As expected, an antibody against the N-terminus of vimentin recognized all constructs. An anti-actin antibody was used as a control (Figure 3B).

### The Differential Exposure of Vimentin Tail Regions Occurs in Several Cell Types

**FIGURE 4:**
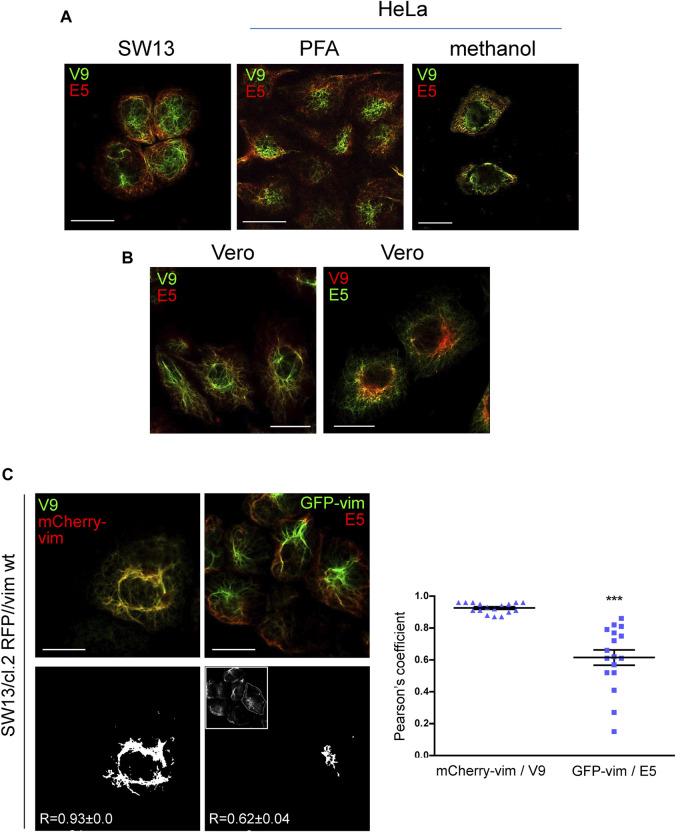
Differential recognition of vimentin C-terminal epitopes in several cell types. **(A)** Vimentin in SW13/cl.2 cells stably expressing RFP//vimentin wt fixed with PFA and in HeLa cells fixed with PFA or methanol, as indicated, was observed by immunofluorescence with anti-vimentin V9-Alexa488 and anti-vimentin E5-Alexa546, followed by confocal microscopy. Single sections at 1–2 microns from the basal plane are shown. **(B)** Vero cells fixed with PFA as above were immunostained with the indicated antibodies, namely: left panel, anti-vimentin V9-Alexa488 plus anti-vimentin E5-Alexa546; right panel, anti-vimentin V9-Alexa568 plus anti-vimentin E5-Alexa488, and observed by confocal microscopy. **(C)** SW13/cl.2 RFP//vimentin wt cells, expressing untagged vimentin, were cotransfected with a small amount of mCherry-vimentin (mCherry-vim) or GFP-vimentin (GFP-vim), as indicated, to highlight the full vimentin network. Subsequently, they were subjected to immunofluorescence with V9-Alexa488 and E5-Alexa546, respectively. The overlap between the total vimentin signal and that of the corresponding antibodies was calculated as the Pearson coefficient from 17 cells per experimental condition. The adjacent graph depicts all values obtained along with the mean values ± SEM. Differences between experiments were significant (***p < 0.0001), as determined by unpaired Student’s t-test. Scale bars, 20 µm.

The results shown above suggest a differential exposure of vimentin epitopes at distinct subcellular locations. For subsequent experiments, we decided to compare signals from the V9 and E5 antibodies because they belong to the same immunoglobulin subtype (IgG_1_, Kappa light chain) and are available as direct fluorescent conjugates. First, we explored whether the differential spatial distribution of V9 and E5-immunoreactivity occurred in several cell types (Figure 4). Vimentin-deficient SW13/cl.2 cells stably transfected with vimentin wt showed a juxtanuclear enrichment of V9-positive vimentin, whereas the recognition by E5 was more defined at the cell periphery (Figure 4A). A preferential detection of central vimentin by V9 and peripheral vimentin by E5 was also observed in HeLa cells, independently of the fixation method (Figure 4A), as well as in Vero cells (Figure 4B). In the latter cell type, a control inverting the conjugated fluorophores, i.e., employing V9-Alexa Fluor 546 and E5-Alexa Fluor 488, confirmed the selective detection of the epitopes at different cellular locations (Figure 4B).

In order to assess this differential distribution by methods not relying only on antibody binding, we combined immunofluorescence with the transfection of a small proportion of fluorescent vimentin constructs to directly light up the vimentin network (Figure 4C), as previously reported (Ho et al., 1998; Yoon et al., 1998; Pérez-Sala et al., 2015). Interestingly, V9-Alexa488 fluorescence markedly colocalized with mCherry-vimentin, thus reflecting protein abundance, whereas E5-Alexa546 fluorescence showed scarce colocalization with GFP-vimentin at the center of the cell, but detected the peripheral areas where the GFP-vimentin signal was less intense. This limited correlation between E5 signal and total vimentin highlights again the restricted accessibility of the 419–438 segment (E5 epitope) in vimentin at the robust central filaments, compared to peripheral regions, where the density of the network is lower.

### High Resolution Analysis of the Differential Immunoreactivity of Vimentin Tail Segments

**FIGURE 5:**
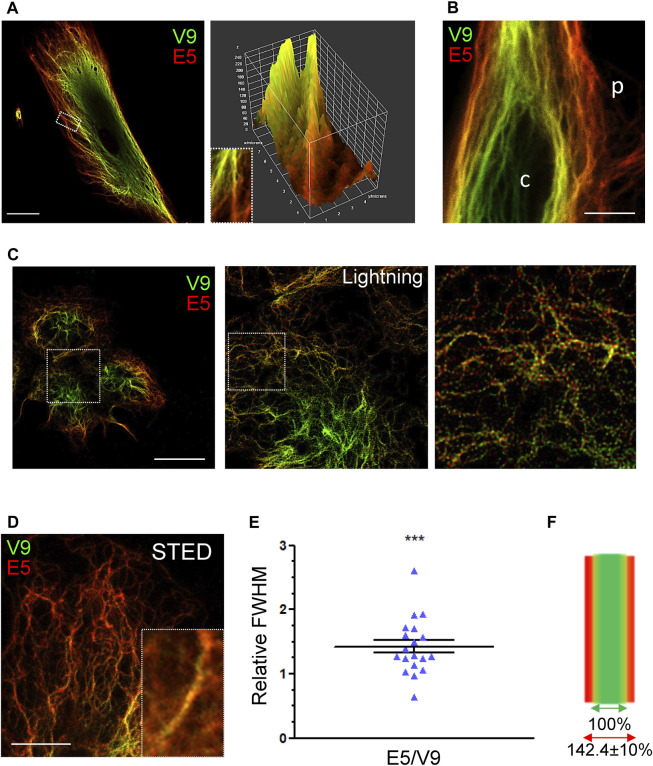
Analysis of the differential accessibility of vimentin tail domain epitopes by high resolution microscopy. **(A)** Human fibroblasts were stained with V9-Alexa488 and E5-Alexa546 antibodies. A single confocal section is shown. The area delimited by the rectangle in the left panel is enlarged in the right panel (inset) and the corresponding surface plot illustrating the graded change in immunoreactivity of the vimentin filaments is shown. Scale bar, 20 μm. **(B)** Human fibroblasts stained as in **(A)** were visualized by STED superresolution microscopy. The peripheral (p) and central regions (c) of the cell are indicated. Scale bar, 5 μm. **(C)** SW13/cl.2 cells stably expressing RFP//vimentin wt were stained with V9-Alexa488 and E5-Alexa546 and visualized by confocal microscopy using an SP8 confocal microscope; the middle and right panels show enlarged areas of images acquired with the lightning module and processed with the lightning deconvolution tool afterwards. Scale bar, 20 µm. **(D)** Insight into vimentin filaments at the cell edge using STED super-resolution microscopy obtained from SW13/cl.2 cells expressing RFP//vimentin wt. Inset depicts a representative filament. Scale bar, 5 μm. **(E)** Scatter plot depicting the relative width of filaments as estimated from the signals of the E5 and V9 antibodies. Width was calculated, for each channel, as full width at half maximum (FWHM) in filaments from STED images (*n* = 19). Results proved significant when mean value was compared to theoretical value (1) through one sample *t*-test (***p < 0.001). **(F)** Graphical representation of vimentin filaments width, as recorded by each antibody, according to data presented in **(E)**. Values are indicated as mean ± SEM.

Next, the differential recognition of the V9 and E5 epitopes was assessed by high resolution microscopy strategies (Figure 5). Analysis of single filaments in fibroblasts by confocal microscopy clearly showed a different proportion of the fluorescence intensities along the filament length, with immunoreactivity changing gradually from a V9-prevalent to an E5-predominant signal towards the distal portion of filaments (Figure 5A). Moreover, a surface plot of the filament confirmed a better accessibility of the E5 epitope towards the filaments tip. A lower accessibility of the E5 epitope in perinuclear filaments was also observed by STED (Figure 5B). Further characterization of vimentin immunoreactivity using the Leica Lightning module allowed visualization of V9 and E5 signals on single filaments in SW13/cl.2 cells expressing vimentin wt, and revealed an alternate dotted pattern with a higher abundance of V9 positive spots towards the center of the cell, and of E5 towards the cell periphery (Figure 5C).

Next, STED microscopy was performed on thin filaments close to the cell periphery (Figure 5D), and the distribution of antibodies on the filament was assessed determining the width of each signal through FWHM (full width at half maximum) measurements, as described in the methods section. Filaments alike in size, at a similar distance from the plasma membrane and with comparable E5 and V9 signal intensities were chosen for quantification. Compared to the width of the V9 signal, the amplitude of E5 staining was 1.42 times larger (1.42 ± 0.10) (Figure 5E), as represented in Figure 5F. Although further confirmation is required, these results could suggest that the E5 epitope is accessible at points farther from the center of the filament, thus, in a more open tail conformation.

### Exposure of Vimentin Tail Segments in a Truncated Protein

**FIGURE 6:**
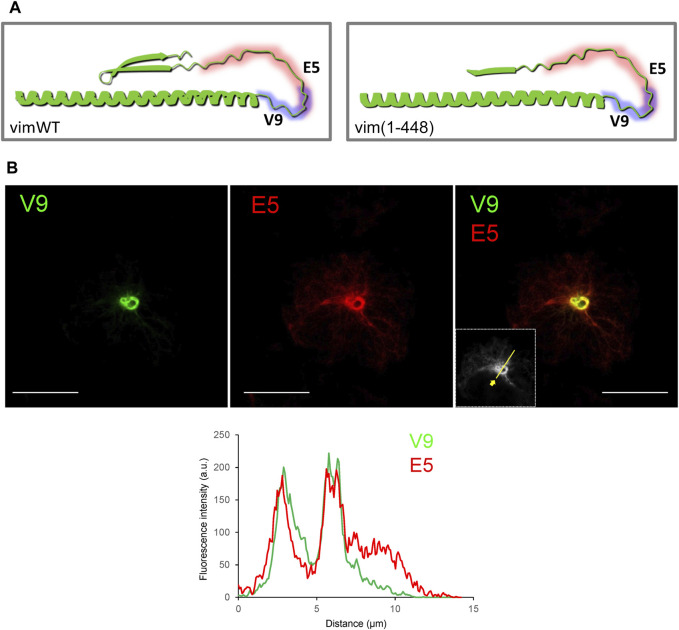
Recognition of a C-terminal truncated vimentin mutant by the V9 and E5 antibodies. **(A)** Models of the vimentin C-terminus, according to AlphaFold predicted structure. Left panel, vimentin residues 350 to 466 are presented illustrating the predicted interaction between the β-turn and the epsilon site, and the resulting loop of the tail domain, potentially masking certain epitopes. Right panel, model for the truncated vimentin (1–448) mutant, lacking the constriction of the interaction, thus potentially allowing exposure of additional segments of the tail. **(B)** Immunoreactivity of a vimentin (1–448) mutant towards V9 and E5 antibodies was assessed by immunofluorescence in SW13/cl.2 transfected with this construct. Results are representative from three experiments with similar results. The fluorescence intensity profiles along the line depicted in the inset are shown in the lower panel. Scale bars, 20 μm.

The vimentin tail has been proposed to form a hairpin structure due to the interaction of the β-turn comprising the RDG motif with the epsilon-site on the distal portion of the rod (Kouklis et al., 1991; Hess et al., 2013). Indeed, a model of the C-terminal segment of the vimentin monomer obtained from AI-based AlphaFold shows that the β-turn-rod interaction could impose a bend of the tail forming a loop that would project from the filament (Figure 6A). The V9 epitope could occupy the apex of the loop, therefore, being accessible in spite of filament compaction or bundling, whereas the E5 epitope could be shielded from recognition in the folded conformation. To explore this possibility we performed immunostaining of a vimentin (1–448) truncated construct, which lacks the RDG motif (Duarte et al., 2019) (Figure 6B). As previously characterized, vim(1–448) yielded a mixed assembly pattern consisting of both extended filaments and curly bundles (Duarte et al., 2019). Vim(1–448) could be detected with both V9 and E5 antibodies. Interestingly, equilibration of maximal intensities showed that the dense swirls were similarly detected by both antibodies. Nevertheless, under these conditions, peripheral filaments were still preferentially detected by E5. This indicates that removal of the RDG motif appears to improve the relative accessibility of the E5 epitope in dense bundles, although its exposure continues to be greater in peripheral filaments.

### Accessibility of Vimentin Tail Segments in Different Cellular Contexts

The results shown above suggest the coexistence of several populations of vimentin in cells, differing in the accessibility of the tail domain under standard cell culture conditions. Therefore, we aimed to explore whether this phenomenon occurred in different cellular situations, where a higher dynamicity of the vimentin network could be expected.

**FIGURE 7:**
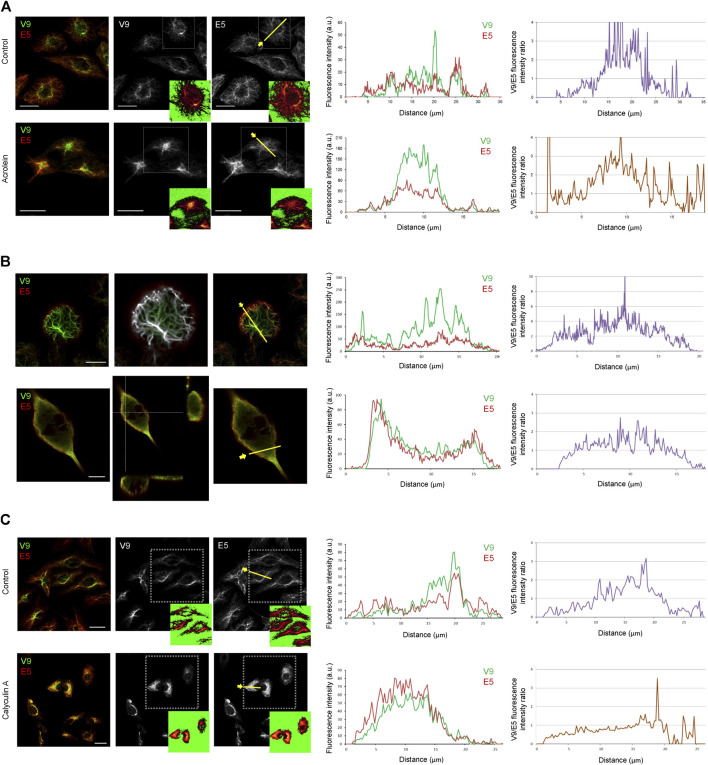
Differential accessibility of vimentin C-terminal epitopes under various experimental conditions affecting vimentin assembly. **(A)** Effect of electrophilic stress on the exposure of V9 and E5 epitopes. Vero cells were treated with vehicle (control) or 10 μM acrolein for 2 h, as indicated, and processed for immunodetection with V9-Alexa488 and E5-Alexa546. Single confocal images of merged and individual channels are shown. Regions of interest, delimited by dotted squares are shown in insets as visualized with the LUT command to illustrate the cell contours and the levels of saturation of the signals. Fluorescence intensity profiles along the yellow line were obtained for each channel and shown at the right. The far right panel shows the intensity ratio of V9 and E5 fluorescence intensities. **(B)** Immunoreactivity of vimentin tail segments in mitotic cells. Upper panels, SW13/cl.2 cells expressing vimentin wt undergoing mitosis were imaged as above and a single confocal section at a basal z-plane is shown. The middle image shows the colocalization mask highlighting the points of coincidence between the signals of both antibodies (in white). Lower panels, human fibroblasts undergoing mitosis were immunostained as above. A single confocal section is shown in the left image and orthogonal projections are depicted in the middle panel to illustrate the peripheral disposition of the E5 signal. Fluorescence intensity profiles along the yellow lines drawn in the right images and the V9/E5 intensity ratio are plotted at the right for both cell types. **(C)** Effect of calyculin A on vimentin tail immunoreactivity. Vero cells treated with vehicle (control) or 20 nM calyculin A for 20 min and stained with V9 and E5 antibodies. Regions of interest, delimited by dotted squares are shown in insets visualized with the LUT command. Fluorescence intensity profiles along the yellow line and V9/E5 fluorescence intensity ratio are plotted at the right. Scale bars, 20 μm.

The vimentin network suffers a drastic reorganization in response to stress, as already illustrated in Figure 1. Therefore, we first explored whether the formation of aggresome-like structures elicited by electrophilic compounds implied changes in the recognition of vimentin tail segments by the V9 and E5 antibodies. Treatment of Vero cells with the electrophilic compound acrolein induced intense vimentin remodeling with formation of thick bundles and aggresome-like structures that could be detected with both V9 and E5 antibodies in a significant proportion of cells (Figure 7A). Interestingly, imaging signals from both antibodies below saturation point revealed the predominance of the V9 signal at the center of aggresomes, and of E5 in peripheral filaments. This is illustrated by the fluorescence intensity profiles as well as by the ratio of V9/E5 fluorescence intensities in the aggresome vs. peripheral filaments, which reaches 3–4 in the aggresomes but is lower in the surrounding cytoplasm (Figure 7A). Since, as shown above, the V9 signal appears to correlate with vimentin abundance, these observations clearly show that V9, but not E5, efficiently detects the accumulation of vimentin in the aggresome.

During mitosis, vimentin is phosphorylated by a series of kinases (Yamaguchi et al., 2005; Izawa and Inagaki, 2006), which results in a cell type-dependent reorganization, characterized by different degrees of disassembly, ranging from filament preservation to nearly complete solubilization (Duarte et al., 2019; Chou et al., 1989). In SW13/cl.2 cells stably expressing vimentin, filaments are preserved in mitosis and translocate to the cell periphery, where they interact with the actin cortex (Duarte et al., 2019). Monitoring the V9 epitope showed its accessibility in the robust filaments that line the cell cortex at the basal layers of mitotic cells (Figure 7B, upper images), as previously reported by us (Duarte et al., 2019). In contrast, the E5 epitope was less accessible in these structures and was more clearly detected at a peripheral cell layer, as illustrated in the fluorescence intensity profiles and the plot of V9/E5 intensity ratios, where values at the cell periphery are below 1. Nevertheless, points of coincidence of the two signals could be spotted (Figure 7B, colocalization mask). In sharp contrast, in mitotic human fibroblasts, vimentin distributed in bundles with a loose appearance or as a diffuse background indicative of disassembly or solubilization, respectively (Figure 7B, lower images). Interestingly, the V9 and E5 signals also displayed obvious differences under these conditions. Both the loose bundles and the internal diffuse background showed slightly predominant V9 immunoreactivity, whereas the E5 epitope was preferentially detected at the most peripheral region. These differences were obvious in orthogonal projections of these images, which showed an external layer with more intense E5 reactivity and the internal volume, positive for both signals, but with areas of preferential V9 staining, as illustrated in the fluorescence intensity profiles and the corresponding fluorescence ratios. These observations indicate that differences in the accessibility of both vimentin tail epitopes persist, in spite of the disassembly occurring in mitosis in this cell type.

Phosphatase inhibitors, like calyculin A, an inhibitor of protein phosphatase 1 (PP1) and 2A (PP2A), have been shown to induce vimentin hyperphosphorylation and solubilization (Quintanar, 2000; Eriksson et al., 2004). Therefore, we explored the effect of calyculin A on the recognition of vimentin by V9 and E5 antibodies (Figure 7C). Incubation with calyculin A elicited diverse morphological alterations of the vimentin network in Vero cells, including filament coalescence into thick bundles and accumulations that displayed a similar recognition by E5 and V9 antibodies, indicating a more similar accessibility of epitopes. These results, illustrated in the fluorescence intensity profiles and the V9/E5 fluorescence ratios shown in Figure 7C suggest that vimentin phosphorylation, as a consequence of phosphatase inhibition, could improve the accessibility of the E5 epitope.

### Exposure of Vimentin Tail Segments in Phosphomimetic Mutants

**FIGURE 8:**
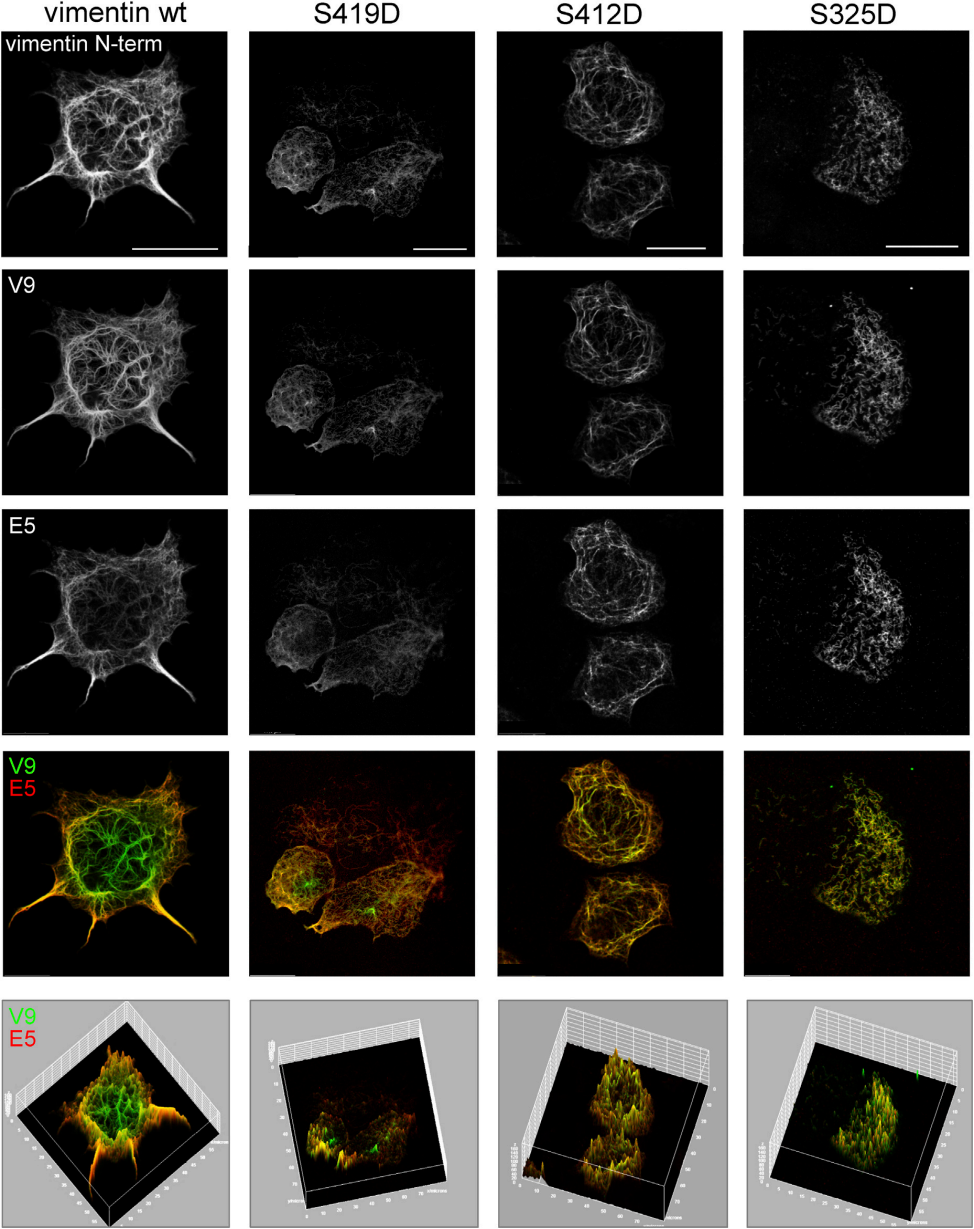
Assembly and C-terminal epitope recognition of vimentin phosphomimetic mutants. SW13/cl.2 cells were transfected with plasmids for expression of vimentin wt or the indicated phosphomimetic mutants. The distribution of the vimentin network was assessed by immunofluorescence with the N-term antibody (N-term-Alexa647), and the immunoreactivity of the tail domain monitored by staining with V9-Alexa488 and E5-Alexa546, as indicated. Single channels and merged V9/E5 images are shown. The bottom panels depict the surface plots for every condition in order to better illustrate the intensity and distribution of V9 and E5 signals. Scale bars, 20 μm.

Interestingly, the vimentin tail displays several phosphorylation sites, although their function is not well understood. As an initial approach to investigate a potential role of phosphorylation in the accessibility of vimentin tail segments we studied several mutants introducing phosphomimetic residues at sites close to the V9 and E5 epitopes, namely, vimentin S419D and vimentin S412D. In addition, a phosphomimetic mutant in segment 2B of the rod, vimentin S325D, was studied for comparison. The impact of these mutations on vimentin network distribution was assessed first by immunofluorescence with an antibody against the N-terminal end of vimentin, in order to avoid potential interferences of the mutations with the binding of antibodies against the tail domain (Figure 8). Vimentin S419D and S412D formed extended filament networks similar to that formed by vimentin wt, although filaments frequently appeared less robust and with a reticular disposition. In contrast, vimentin S325D formed apparently shorter filaments of irregular orientation. Immunofluorescence of these mutant networks with the V9 and E5 antibodies showed an attenuation of the differences in the recognition patterns. In vimentin S419D, the central area of the cell was still preferentially recognized by V9. In contrast, vimentin S412D showed a higher overlap of V9 and E5 signals, indicating a more similar accessibility of the corresponding epitopes. Remarkably, vimentin S325D filaments appeared to be comparably stained by V9 or E5, irrespectively of the cell region. These observations, which are illustrated in the surface plots (Figure 8, bottom panels), suggest that introducing phosphomimetic residues at several sites of the vimentin C-terminus, impacts its assembly and/or the disposition of the tail domain, thus influencing exposure of different segments. Nevertheless, other explanations are possible as it will be discussed below.

## Discussion

The intermediate filament protein vimentin can form an amazing variety of dynamic interchangeable structures, which are involved in a plethora of cellular functions. Vimentin assembly, location and interaction can be finely tuned by posttranslational modifications (PTM), which can occur throughout the sequence of the protein giving rise to a wide variety of proteoforms (Snider and Omary, 2014; Griesser et al., 2021). The disordered tail of vimentin, which can harbor a variety of PTM (Griesser et al., 2021), has been reported to modulate the assembly of filaments and to support interactions with other cytoskeletal structures (Kouklis et al., 1993; Duarte et al., 2019). Nevertheless, the conformation of this segment is not known and could be affected by several factors including PTM, binding of divalent cations and/or protein-protein interactions (Lin et al., 2010). Here, we have observed that the accessibility of various segments of vimentin C-terminus displays defined spatial patterns in cells, which suggests the presence of distinct vimentin populations differing in the conformation of the tail domain, with a selective subcellular distribution.

The first indication of a differential exposure of tail segments was obtained when comparing the signals from GFP-vimentin and from vimentin detected by immunofluorescence with antibodies against the N- or C-terminus of the protein upon treatment with HNE, an electrophilic lipid which causes filament retraction towards the nucleus. Unexpectedly, whereas, apparently, GFP-vimentin and the anti-N-terminus antibody did not reveal vimentin filaments at the periphery of HNE-treated cells, the C-term antibody did (Figure 1). Interestingly, approaches combining immunological detection and fluorescent vimentin constructs (Figure 1E) clarified that these differences arose from the fact that the intense GFP-vimentin and anti-N-terminus signals at the HNE-induced bundles became saturated before the scarce peripheral filaments became detectable. Conversely, as the C-term antibody poorly recognized vimentin bundles and accumulations, the signal of peripheral vimentin could be detected without reaching saturation.

Interestingly, detailed characterization of vimentin tail immunoreactivity has unveiled a differential accessibility of two close vimentin tail segments, namely, the V9 and E5 epitopes, at distinct cell regions. The V9 epitope, located in segment 411–423, is accessible throughout the filament network, whereas the E5 epitope, located between residues 419 and 438, is shielded from recognition in bundles at the cell center and in aggresome-like structures, but is preferentially detected in sparse filaments. In this case, combination of immunological detection and fluorescent vimentin constructs clearly showed that the V9 signal keeps a better correlation with the overall distribution and abundance of vimentin throughout the cell, and therefore, reflects more accurately the morphology of the network, whereas E5 immunodetection poorly recognizes robust filaments under resting conditions. This reduced accessibility of the E5 epitope could be due to several reasons including the density of filament packing, a differential conformation of the vimentin tail, presence of PTM and/or protein interactions, without disregarding intramolecular or intrafilament interactions, for instance with the head domain.

Earlier works have described the distinct subcellular localization of vimentin proteoforms. Phosphorylated disassembled vimentin has been located at leading lamelipodia (Helfand et al., 2011) and vimentin phosphorylated at S459 has been reported to be mostly located at cell protrusions (Kotula et al., 2013). In turn, vimentin bearing certain oxidative PTM has been described to be destined for secretion to the extracellular medium or exposure at the plasma membrane (Frescas et al., 2017). In this work, we observe a subpopulation of vimentin forming robust filaments at the center on the cells, mainly underneath the nucleus in resting cells and at the center of basal planes in mitotic cells that are selectively stained with the V9 antibody, with poor accessibility of the E5 epitope.

Although the structure of the vimentin tail is not known, detailed *in vitro* work indicates the existence of “points of order” in this intrinsically disordered domain (Hess et al., 2013). As mentioned before, studies with anti-idiotypic antibodies (Kouklis et al., 1991) have defined the so-called “beta” and “epsilon” sites, located in the tail, residues 444–452, and at the C-terminal end of the rod, residues 364–416, respectively, the beta site being important for vimentin filament assembly and architecture (Kouklis et al., 1993; Makarova et al., 1994). Moreover, the beta site has been proposed to fold over the epsilon site in assembled vimentin, which would lead to the formation of a loop, hypothetically protruding from filaments.

**FIGURE. 9:**
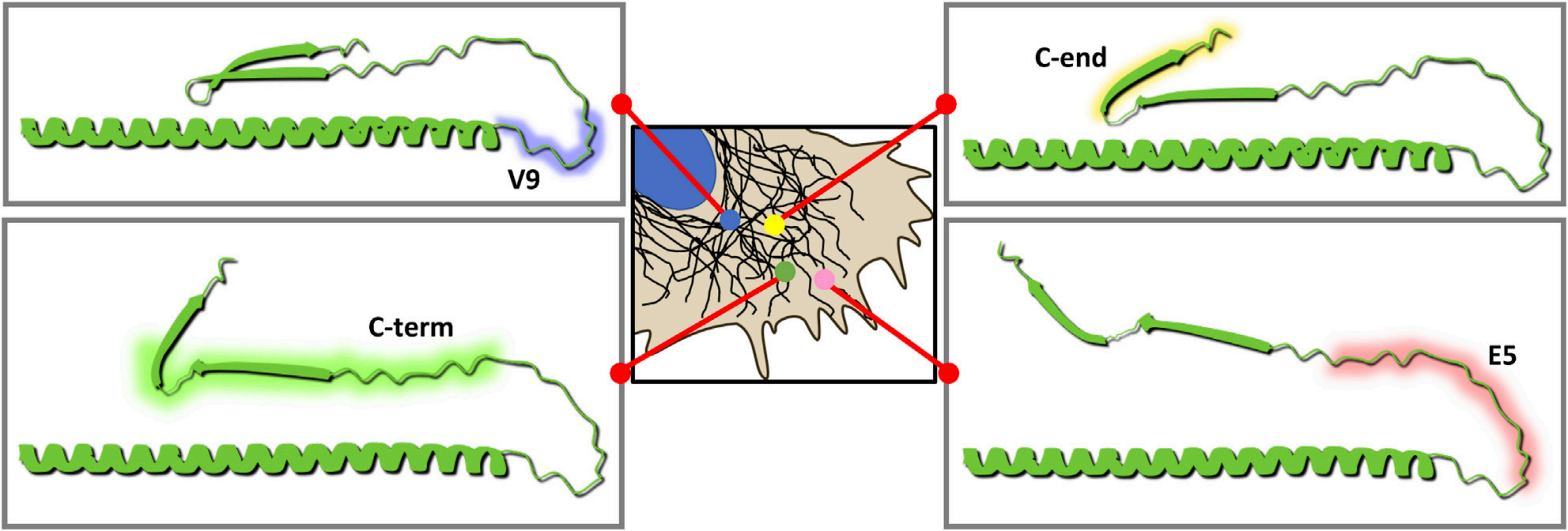
Model of the potential conformations of vimentin tail based on the differential exposure of epitopes observed. Vimentin is represented from the residue 350 to the C-terminus. The structure depicted here is based on the predictions of vimentin structure available in AlphaFold. Antibody epitopes are indicated in a color code as in Figure 2: V9 in blue, E5 in pink, “C-term” in green and “C-end” in yellow. The areas of the cell where epitopes are preferentially detected in each case are indicated, similar to Figure 2. Closer to the cell periphery, where vimentin would adopt the most extended conformation, all antibodies would be putatively capable of binding their epitopes. The E5 epitope is the first one to be less recognizable when approaching the cell center, followed by “C-term.” In areas closer to the nucleus, V9 antibody would be the antibody to preferentially bind vimentin, putatively in its most compact conformation.

Together with these previous works, our results could propose a model in which the V9 epitope, centered in N417, would protrude from assembled filaments, therefore being accessible in compact filaments and even bundles, whereas the E5 epitope, closer to the beta-turn, could be shielded in robust filaments due to the β-turn-epsilon site interaction and/or to the apposition of vimentin tail pairs occurring during polymerization. This would be particularly important under certain stress conditions causing further filament condensation, which could mask the C-terminal epitopes to make them virtually undetectable as shown in Figure 1. A schematic representation of hypothetical conformations of the vimentin tail, as suggested from the exposure of the various epitopes, and assisted by AlphaFold, is presented in Figure 9. Interestingly, it is well known that tail domains of other intermediate filaments, for instance neurofilaments, are involved in regulation of their assembly or crossbridging, in a process that is modulated by phosphorylation occurring selectively at distinct cellular locations (Yuan et al., 2012). In addition, the conformation of tail domains can be differentially involved in protein-protein interactions. The tail domain of desmin, which is 71% homologous to that of vimentin, bears important sites for interaction with the chaperone αB-crystallin (Sharma et al., 2017). In reconstituted systems, αB-crystallin has been shown to bind to desmin oligomers and coassemble with them, but to scarcely bind to preformed filaments. Thus, αB-crystallin has been proposed as a sensor for different assembly states of desmin (Sharma et al., 2017). In an analogous way, the results shown here could indicate that certain antibodies against the C-terminal domain, mainly E5 and C-term, may serve as “sensors” or indicators of vimentin assembly. In line with our results, early studies on desmin also described a differential accessibility of a C-terminal segment. In particular, the region containing the RDG motif, residues 442–450 in chicken desmin, was accessible to site-specific antibodies in disassembled subunits and loosely packed filamentous structures, but not in desmin mature filaments, neither *in vitro* nor in muscle cells (Birkenberger and Ip, 1990). Therefore, these findings support our interpretation regarding the different immunoreactivity of vimentin tail segments depending on the assembly state of the protein.

Importantly, epitope exposure, and therefore tail conformation, could be regulated by PTM either by affecting the tail per se or indirectly by modulating the assembly state of vimentin. Indeed, it has been previously noted that the beta site is flanked by phosphorylation sites, including S430, S458 and S459 (Hess et al., 2013; Kornreich et al., 2015) that influence vimentin assembly (Eriksson et al., 2004).

Notably, EPR studies of *in vitro* PKA phosphorylated vimentin showed an important impact of phosphorylation on the C-terminal region of the rod (Pittenger et al., 2008). S412, S419 and S420 were found to be phosphorylated in this study, although the precise impact of these sites was not assessed. Indeed, although vimentin phosphorylation is widely recognized to control vimentin assembly, modifications at the head domain are much better characterized both from the mechanistic and functional points of view. The vimentin head is also known to fold over the rod forming a loop-like structure. Interestingly, EPR studies have shown that the phosphorylation of the head domain laterally separates the heads but does not necessarily disrupts the head-rod association (Aziz et al., 2009). Our observations do not allow discerning whether shielding of the E5 epitope is due to lateral apposition or to the tail-rod interaction. Nevertheless, our results with the 1–448 truncated vimentin mutant and high resolution microscopy indicate that likely both the beta-epsilon interaction and lateral apposition of the tails are involved in shielding the E5 (and/or C-term) epitopes in cells, since removal of the β-site partially improves the detection of the E5 epitope in bundles.

In contrast, differences in the exposure of the V9 and E5 epitopes persist in mitotic cells, even in spite of the apparent disassembly of vimentin in mitotic fibroblasts. This may indicate that disassembly in mitosis does not necessarily imply marked changes in tail conformation. Conversely, we have observed that calyculin A, which is known to increase the phosphorylation state of vimentin by precluding dephosphorylation, induces the formation of cellular vimentin assemblies with a more comparable exposure of V9 and E5 epitopes. Interestingly, calyculin A has been reported to increase phosphorylation at sites already modified in interphase cells, including some present in the C-terminal domain, and different from those that are phosphorylated in mitosis (Eriksson et al., 1992; Eriksson et al., 2004). Several tail domain residues have been found phosphorylated in mitosis, although to a lower extent and apparently exerting a weaker functional impact than those present in the head domain (Chou et al., 2003). Thus, the importance of phosphorylation on vimentin tail conformation could be site-dependent.

In fact, our observations with single phosphomimetic mutants suggest a variable impact of the phosphorylation of certain sites on network organization, which appears to be more intense when the mutated sites are closer to or within the rod domain. Under these conditions, parallel changes in the degree of assembly and vimentin tail epitope exposure appear to take place, with mutations at the beginning of the tail (S412D), and above all, at the C-terminal region of the rod domain (S325D), leading to sparser filaments with a similar exposure of epitopes and loss of the cell region dependence of the tail conformation. In contrast, the pattern of the S419D mutant is closer to the wt. Therefore, it could be hypothesized that charged residues and/or phosphorylation at certain positions could impact the conformation of the tail domain. Whether this potential conformational change implies unfolding or unpairing of tail segments remains to be elucidated.

According to the PhosphoSite database, both S412 and S419 have been found phosphorylated in ischemic tumors (Mertins et al., 2014) and in HeLa cells after treatment with gambogic acid (Yue et al., 2016). In addition, phosphorylation of S419 has been found in BHK21 fibroblasts treated with calyculin A (Eriksson et al., 2004), whereas phosphorylation of S412 was shown in cells arrested in mitosis by nocodazole treatment (Olsen et al., 2010). In turn, phosphorylation of S325 has been described in multiple cell types and experimental conditions (https://www.phosphosite.org/siteAction.action?id=4148959). Interestingly, the proximity of S325 to C328, a hot spot for modification by oxidants and electrophiles and a key regulator of vimentin assembly and reorganization in response to oxidative stress, raises the possibility of cross-talk between S325 phosphorylation and C328 modification in the regulation of vimentin organization, which deserves further study.

In summary, our results point to the existence of diverse vimentin populations with different tail conformations in cells. These populations can be distinguished by the exposure of precise epitopes and likely reflect distinct states of assembly, potentially regulated by PTM, including phosphorylation. These different populations could also be involved in distinct interactions or functions which deserve further investigation.

## Data Availability

The raw data supporting the conclusion of this article will be made available by the authors, without undue reservation.
